# Design and Fabrication of a Wavelength-Selective Near-Infrared Metasurface Emitter for a Thermophotovoltaic System

**DOI:** 10.3390/mi10020157

**Published:** 2019-02-25

**Authors:** Atsushi Sakurai, Yuki Matsuno

**Affiliations:** Niigata University, Niigata 950-2181, Japan; t11m063b@gmail.com

**Keywords:** near-infrared light, wavelength-selective emitter, metasurface, photolithography method

## Abstract

In this study, a tungsten-SiO_2_-based metal–insulator–metal-structured metasurface for the thermal emitter of the thermophotovoltaic system was designed and fabricated. The proposed emitter was fabricated by applying the photolithography method. The fabricated emitter has high emissivity in the visible to near-infrared region and shows excellent wavelength selectivity. This spectral emissivity tendency agreed well with the result calculated by the finite-difference time-domain method. Additionally, the underlying mechanism of its emission was scrutinized. Study of the fabrication process and theoretical mechanisms of the emission, clarified in this research, will be fundamental to design the wavelength-selective thermal emitter.

## 1. Introduction

Recently, with global warming and the exhaustion of fossil fuels, there is an urgency to adapt an efficient way of utilizing renewable energy. Among the numerous renewable energies, solar energy is one of the most promising candidates. As an efficient method to convert solar energy into electricity, the thermophotovoltaic (TPV) system is attracting increasing attention [1,2]. The TPV system can efficiently convert thermal energy from any kind of heat source into electricity by absorbing the incident thermal energy with a wavelength selective absorber and emitting that energy with a thermal emitter, which emits photons in a certain wavelength that matches the highest quantum efficiencies (QE) of a specific photovoltaic (PV) cell. Moreover, the TPV system is environmentally friendly and discharges hardly any harmful substances or noise.

In the TPV system, the wavelength-selective thermal emitter plays a significant role in its energy efficiency [3]. The ideal thermal emitter requires high emissivity at the wavelength that matches the highest QE of a PV cell, and low thermal emissivity in the infrared region to reduce radiative heat loss. Additionally, its emissivity should be independent from the polarization and incident angles. To obtain a high-efficiency emitter, a metal–insulator–metal (MIM)-structured metasurface is employed for this study. MIM metasurfaces have been successfully developed for tailoring thermal radiation [4,5,6,7,8]. Conventional MIM metasurfaces were designed for room temperature applications. Therefore, MIM metasurfaces are usually fabricated with noble metals. But recent studies successfully demonstrated MIM metasurfaces for high temperature applications [9,10]. Tungsten (W) is a promising material for high temperature applications because it has a high melting point. However, the previous studies of such MIM metasurfaces with W were very few because of the difficulty in patterning W nano patterning. Lin et al. [11] computationally designed a W-based anisotropic metamaterial for a broadband absorber, but the designed nano-structure was very complicated. Lefebvre et al. [12] reported the experimental realization of complementary metal-oxide-semiconductor (CMOS)-compatible MIM perfect absorbers, of which their target wavelength is in the mid-infrared range. Therefore, it is necessary to improve the fabrication technology and the optical characteristics of near-infrared metasurfaces for high temperature applications.

In this study, we computationally design and experimentally fabricate a near-infrared metasurface emitter for a GaSb PV cell. To unravel the underlying resonance mechanisms and evaluate the measurement results, electromagnetic (EM) simulations based on the finite-difference time-domain (FDTD) method and an LC-circuit model are performed and compared with the measurement results.

## 2. Computational Design and Experimental Process

**Figure 1:**
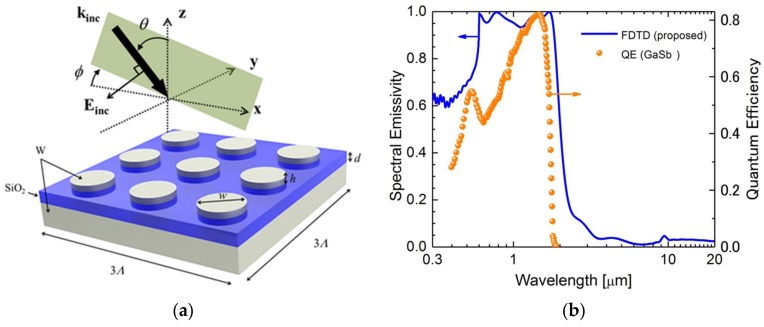
(**a**) Schematic of the proposed metasurface. (**b**) Simulated spectral emissivity of the proposed emitter obtained from finite-difference time-domain (FDTD) simulation (blue dot) and the quantum efficiencies (QE) of the GaSb PV cell (orange dot) [14].

Figure 1a shows a schematic of the proposed emitter (i.e., a periodic metallic disk pattern on a dielectric film, which is placed on top of a metallic film). *θ* and *ϕ* represent the incident and polarization angles of the incident light, respectively. SiO_2_ was chosen as a dielectric spacer and W was chosen as the metallic part of the proposed metasurface. Although there is a mismatch in the thermal expansion coefficient between W and SiO_2_, a metasurface using these materials, which is stable up to 800 K, has been fabricated previously and reported [9]. Therefore, we assumed that our proposed MIM-structured metasurface was reasonable, to be utilized, for the TPV emitter, which requires a high operating temperature. Periods of the unit cell for the *x* and *y* directions were *λ* = 600 nm and the diameter of the W disks was *w* = 350 nm. The height of the W disks and thickness of the dielectric spacer were fixed to 50 and 100 nm, respectively. To compute the proposed structure’s optical characteristics, we employed the Lumerical FDTD software. Dielectric functions of SiO_2_ and W were obtained from the tabulated data from Palik [13].

Figure 1b represents the spectral emissivity calculated from normal reflectance obtained from Lumerical FDTD software (blue dot) for the proposed metasurface and the QE of the GaSb PV cell (orange dot) [14]. Here, since the W substrate is opaque, spectral emissivity (*ε*_λ_) could be calculated from the Kirchhoff’s law (i.e., $\varepsilon_{\lambda }=1-R_{\lambda }$, where $R_{\lambda }$ is the spectral reflectance). It can be seen that the higher emissivity spectrum matched the higher QE region.

**Figure 2:**
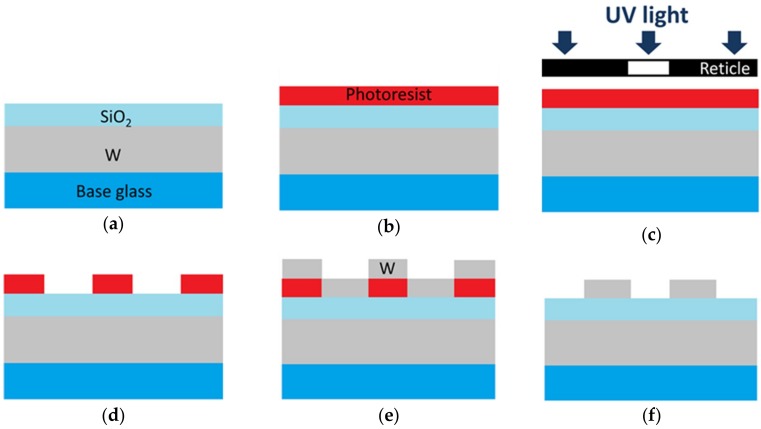
Schematic of the fabrication process of the proposed metasurface. (**a**) Thin W and SiO_2_ films were sputtered; (**b**) photoresist coating; (**c**) ultraviolet (UV) light exposure through a reticle; (**d**) the photoresist layer was developed to form a resist pattern; (**e**) the thin W layer was sputtered; (**f**) lift-off (photoresist with the unnecessary W removed). Reproduced with permission from [8], published by OSA Publishing, 2017.

Figure 2 shows the fabrication process of the proposed metasurface; it was composed primarily of six steps: (a) Thin W and SiO_2_ films were sputtered on a Si plate; (b) coating the top SiO_2_ film with a positive photoresist; (c) pattern transfer to the photoresist with the exposure method using an i-line stepper; (d) after the post exposure bake, the photoresist layer was developed to form a resist pattern; (e) W was sputtered on the sample; and (f) the rest of the photoresist was removed.

Both the base W film and SiO_2_ film were sputtered for 100 nm on the substrate. Then, a positive type photoresist was spin-coated on the top SiO_2_ film and prebaked before exposure to ultraviolet (UV) light to form the resist pattern. Reticle with a 0.375 μm hole was used for the i-line stepper. Afterwards, the exposed photoresist was dissolved and resist patterns appearred. Finally, W was sputtered for 50 nm on the sample and the unnecessary W of the rest of the photoresist was removed by acetone.

A fourier transform infrared spectroscopy (FTIR) spectrometer (Thermo Fisther Scientific iS50 FTIR Nicolet, Thermo Fisther Scientific, Waltham, MA, USA) and a visible spectrometer (Portable Spectral Solar Absorptance Measurement System PM-A2, Kouei Inc., Tokyo, Japan) were used to measure reflectivity for the infrared region and visible region, respectively. Once the reflectivity was obtained, the spectral directional emissivity could be obtained by applying Kirchhoff’s law.

## 3. Results and Discussion

**Figure 3:**
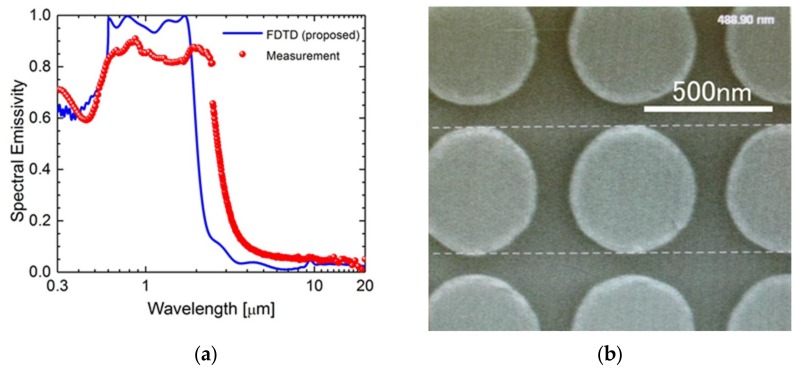
(**a**) Spectral emissivity of the proposed emitter obtained from FDTD simulation (blue line) and the fabricated sample (red dot). (**b**) Scanning electron microscopy (SEM) images of the fabricated metasurface from the top.

To evaluate the fabricated metasurface, its spectral emissivity was measured and compared to the calculation results of the FDTD method. Figure 3a represents the simulated spectral emissivity and the measured spectral directional emissivity of the fabricated metasurface (red dots). A visible spectrometer was utilized for the emissivity measurement up to 2.5 μm and FTIR was applied for the wavelength longer than 2.5 μm. The measured spectral emissivity showed reasonable agreement with the simulated spectral emissivity. However, there was an emissivity mismatch at the wavelength shorter than the cut-off wavelength. Figure 3b displays the top scanning electron microscopy (SEM) images of the fabricated metasurface. It can be seen that the W disks showed excellent symmetry. However, the diameters of the W disks were patterned slightly larger than as designed. This was the reason why the cut-off wavelength was red-shifted. In our future work, the pattern transfer technique will be improved.

**Figure 4:**
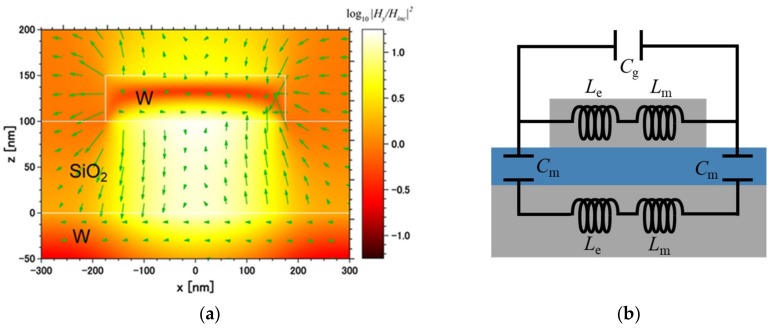
(**a**) Electromagnetic (EM) field profiles of the proposed emitter at 1.7 μm, calculated by FDTD simulation. The color counter shows the logarithm of the normalized magnitude of the square of the *y*-component magnetic field and the vectors show the direction and magnitude of the electric field; (**b**) equivalent LC circuit model.

Since the proposed emitter consists of a MIM structure, magnetic polariton (MP) could be excited in the dielectric spacer between the metals [15,16,17]. The emissivity peak at 1.7 μm, located near the highest QE region, was caused by the excitation of MP. Figure 4a shows the electromagnetic (EM) field distribution at the emissivity peak along the *x–z* plane at *y* = 0 nm. The color contour shows the logarithm of the normalized magnitude of the square of the *y*-component magnetic field. The vectors show the direction and magnitude of the electric field. At the peak wavelength, highly localized *y*-component magnetic field enhancement can be observed in the SiO_2_ film between the W disk and the bottom W plate. Furthermore, the electric field created a closed current loop, which created an enhanced magnetic field and thus formed MP. Therefore, the proposed emitter—which excites MP—could be said to have a strong emissivity peak at the highest QE wavelength region of the PV cell.

As the theoretical method to predict the MP resonant wavelength, a LC circuit model can be applied to the proposed metasurface. Figure 4b describes the LC circuit model applied for the proposed emitter structure. Here, inductance and capacitance are represented as follows [15,16,17]:

(1) $$L_{e,W}=-\frac{w}{\varepsilon_{0}\omega^{2}w\delta_{W}}\frac{\varepsilon_{W}^{\prime }}{\left({\varepsilon^{\prime }}_{W}^{2}+{\varepsilon^{\prime \prime }}_{W}^{2}\right)}$$

(2) $$L_{m,W}=\frac{1}{2}\mu_{0}d$$

(3) $$C_{g,W}=\frac{\varepsilon_{0}hw}{\Lambda -w}$$

(4) $$C_{{m,\mathrm{SiO}}_{2}}=c_{1,W}\varepsilon_{{\mathrm{SiO}}_{2}}^{\prime }\varepsilon_{0}\frac{w^{2}}{d}$$

$L_{e,W}$ and $L_{m,W}$ are the kinetic and magnetic inductance of W. $C_{g,W}$ is used to approximate the gap capacitance between the W disks. $C_{{m,\mathrm{SiO}}_{2}}$ means the parallel-plate capacitance. $\mu_{0}$ is the permeability of the vacuum and ω is the angular frequency. $\varepsilon_{W}^{\prime }$ and $\varepsilon_{W}^{\prime \prime }$ represent the real and imaginary parts of the dielectric function of W. $\varepsilon_{0}$ and $\varepsilon_{{\mathrm{SiO}}_{2}}^{\prime }$ are the dielectric function of the vacuum and SiO_2_, respectively, and $c_{1,W}=0.32$ is the numerical factor to consider the fringe effect or non-uniform charge distribution along the surface of the capacitor. Originally, the numerical factor is recommended to be used in the range between about 0.2 and 0.3 [15]. $\delta_{W}=\frac{\lambda }{2\pi \kappa_{w}}\;$is the effective penetration depth of W, where $\kappa_{W}$ is the extinction coefficient of W. The total impedance of this LC circuit model can be obtained as:

(5) $$Z_{\mathrm{tot}}\left(\omega \right)=\frac{L_{m,W}+L_{e,W}}{1-\omega^{2}C_{g,W}\left(L_{m,W}+L_{e,W}\right)}-\frac{2}{\omega^{2}C_{{m,\mathrm{SiO}}_{2}}}+L_{m,W}+L_{e,W}$$

Here, note that the dielectric function and penetration depth were wavelength-dependent, and the impedance was a module (i.e., no possible dephasing effects were considered). The resonance conditions of MP could be obtained by zeroing the total impedance (i.e.,$Z_{\mathrm{tot}}=0)$. For the proposed emitter, its resonant wavelength was predicted as 1.7 μm. This predicted wavelength was reasonably close to the peak wavelength of the designed emitter.

The spectral emissivity at the oblique incident waves, up to 30° for Transverse Magnetic (TM) and Transverse Electric (TE) waves, was calculated. The spectral emissivity can be obtained from Kirchhoff’s law (i.e., $\varepsilon_{\lambda }=1-R_{\lambda })$, where the reflectance R was calculated by the FDTD. To clarify the angle-dependent behavior, the wideband analysis at oblique incidence was performed by using the FDTD algorithm suggested by Liang et al. [18].

**Figure 5:**
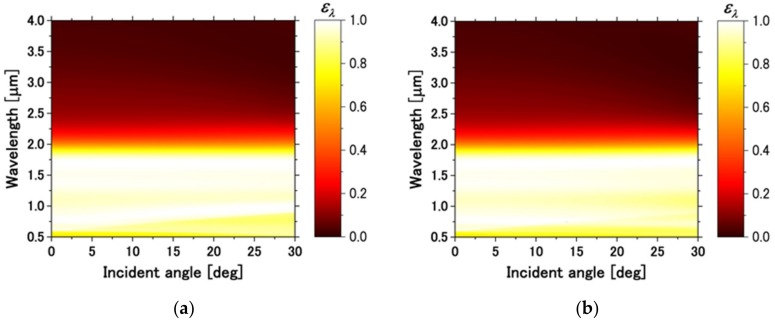
Contour diagram of the emissivity of the proposed emitter obtained by FDTD simulation for (**a**) Transverse Magnetic (TM) waves and (**b**) Transverse Electric (TE) waves, in terms of wavelength and incident angle up to 30°.

The insensibility of the spectral emissivity to the incident and polarization angles was essential for the efficient wavelength-selective emitter. Figure 5 is the contour plot of the spectral emissivity of the proposed emitter obtained from FDTD simulation: (a) at TM waves; and (b) at TE waves, in terms of wavelength and incident angle. It can be observed that for both TM and TE waves, the proposed emitter maintained high emissivity at the high QE wavelength region, while emissivity in the infrared region was low in the wide incident angles. The emissivity peak around 1.7 μm originated from MP. On the other hand, the emissivity peak around 0.7 μm was because of the excitation of surface plasmon [17]. It can be seen that surface plasmon had a directional dependence, but the MP resonance frequency was insensitive to the polar angle. 

**Figure 6:**
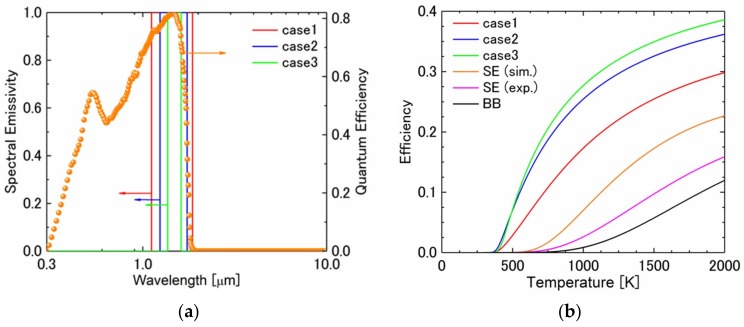
(**a**) Ideal emissivity spectra with wavelength ranges of 0.25 μm (case 1), 0.50 μm (case 2), and 0.75 μm (case 3). The peak position was located at the highest quantum efficiency region. (**b**) Efficiency of the present TPV system with emissivity spectra based on the three cases, the measured and simulated emissivity, and blackbody.

Finally, ideal efficiency of the TPV system, as a function of PV cell temperature, was predicted based on theoretical calculation [19] and available experimental data. Figure 6a shows the ideal emissivity spectra with wavelength ranges of 0.25 μm (case 1), 0.50 μm (case 2), and 0.75 μm (case 3). Here, the peak position was located at the highest quantum efficiency region. For comparison, the present emissivity spectra, $\varepsilon_{\lambda }$ obtained by the numerical simulation, the experimental measurement, and blackbody were used to predict the efficiency of the TPV system.

The input power, *P*_in_, was defined by using Planck’s spectral distribution of emissive power.

(6) $$P_{\mathrm{in}}=\int \frac{2\pi \varepsilon_{\lambda }hc^{2}}{\lambda^{5}\left(\exp[\frac{hc}{\lambda kT}\right]-1)}d\lambda$$

where *h* is the Planck constant, *c* is the speed of light, λ is wavelength, and *k* is the Boltzmann constant. The maximum power *P*_m_ is defined in a manner similar to a classic derivation by Loferski [20].

(7) $$P_{m}=\frac{qJ_{sc}{\left(V_{mp}\right)}^{2}}{\left(kT+qV_{mp}\right)}$$

where *q* is the electron charge, *J*_sc_ is the short-circuit current, and *V*_mp_ is the voltage at maximum power. In the ideal case, we assumed that there was no heat loss for the input power, and the view factor from the emitter to the PV cell was equal for unity. Therefore, the efficiency of the TPV system was defined as:

(8) $$\eta =\frac{P_{m}}{P_{\mathrm{in}}}$$

Figure 6b show the efficiency of the present TPV system with emissivity spectra based on the three cases, the measured and simulated emissivity, and blackbody. The wavelength-selective emitter (SE) had better results than the blackbody (BB) emitter, because the blackbody emitter had a large, undesired thermal radiation in the mid-infrared range. By the simulated emissivity spectra of the SE, when the emitter temperature exceeded about 1600 K, the efficiency approached 20%. However, the efficiency with the experimentally demonstrated SE was about half of the simulated one, because there was a mismatch between the emissivity spectra with the experiment and the quantum efficiency. Next, the three ideal emissivity spectra were introduced to show the theoretical maximum efficiency of this system. In case 1, the wavelength range was the narrowest, hence, the maximum efficiency was expected. From the calculated results, the efficiency of case 1 approached 40% when the emitter temperature became about 2000 K. In case 2 and 3, with broader wavelength ranges, the efficiency decreased, as compared with case 1. It was also true that there was emissivity mismatch with high quantum efficiency area. 

## 4. Conclusion

The MIM-structured W-SiO_2_-based metasurface emitter for the TPV system, which shows a high emissivity peak at the high QE wavelength region of the PV cell, was designed and fabricated. The designed metasurface was fabricated by applying the photolithography method and it was compared to FDTD simulation results. The fabricated metasurface showed high emissivity in the visible to near-infrared light region and indicated excellent wavelength-selectivity. Its emissivity tendency agreed reasonably well with the FDTD simulation. In addition, we clarified that the emissivity enhancement that is underpinning the emissivity peak at 1.7 μm originated from the excitation of MP by calculating the EM field and LC circuit model. It has been shown that the spectral absorption of the proposed emitter is nearly independent of the incident and polarization angles. This study will not only help to understand the mechanisms that can be used to tailor the emissivity enhancement, but will also facilitate the design and practical fabrication of nanostructures for applications in TPV systems.
